# Pulmonary Infections in ICU Patients Without Underlying Disease on Ventilators

**DOI:** 10.5812/traumamon.15958

**Published:** 2014-08-01

**Authors:** Reza Ghanbarpour, Masoud Saghafinia, Mahdi Ramezani Binabaj, Seyed Jallal Madani, Davood Tadressi, Mohammad Javad Forozanmehr

**Affiliations:** 1 Trauma Research Center, Baqiyatallah University of Medical Sciences, Tehran, IR Iran; 2 Students’ Research Committee, Baqiyatallah University of Medical Sciences, Tehran, IR Iran; 3 Anesthesia Group, Faculty of Medicine, Baqiyatallah University of Medical Sciences, Tehran, IR Iran; 4 Faculty of Nursing, Baqiyatallah University of Medical Sciences, Tehran, IR Iran

**Keywords:** Pneumonia, Ventilator-Associated, Intensive Care Units, Ventilators, Mechanical

## Abstract

**Background:**

At present, the use of ventilator support is an important part of treatment in ICU patients. However, aside from its well-known advantages, the use of these devices is also associated with complications, the most important of which is pulmonary infection (PI). PI has a high rate of morbidity and mortality.

**Objectives:**

This study aimed to evaluate the prevalence of PI in mechanically-ventilated patients and the role that factors, such as age, sex, and duration of intubation, play in this regard.

**Materials and Methods:**

This descriptive cross-sectional study evaluated the prevalence of PI in mechanically ventilated patients, with no underlying condition which could compromise their immune system. Age, sex, and duration of intubation were assessed. Data were analyzed using SPSS (version 16) software.

**Results:**

A total of 37 ICU patients on ventilators were evaluated, including 21 males (56.8%) and 16 females (43.2%). The mean age of the patients was 54 ± 19 years (range 19 to 86 years), with a mean age of 52 ± 20 years in men, and 56 ± 18 years in women (P = 0.52). The mean duration of ventilation was 6 ± 4 days (range 2 to 20 days). The mean duration of ventilation was 5 ± 2 days in men, and 6 ± 5 days in women (P = 0.42). A total of 16 patients (43.2%) developed ventilator-associated pneumonia (VAP); of whom, 50% were male and 50% female (P = 0.46). Patients who developed a pulmonary infection had a significantly longer duration of ventilation. The mean duration of ventilation was 8 ± 4 days in patients who had developed VAP, while this duration was 4 ± 2 days in the non-affected patients (P = 0.005). Overall, 17 patients died, and 7 of these deaths were attributed to VAP.

**Conclusions:**

The prevalence of VAP in this study was approximately 43%, which is relatively high. In total, the percentage of deaths due to VAP among the patients was 18.91%. Duration of ventilator support was significantly correlated with the prevalence of PI.

## 1. Background

At present, mechanical ventilation is an important part of the treatment of patients admitted to the intensive care unit (ICU). Considering the fact that patients requiring ventilator support are usually in a critical condition, the risk of hospital infections, especially pulmonary infections following intubation, is extremely high and the rate of morbidity and mortality in these patients sometimes reaches 50 to 70% (1-4). On the other hand, the use of catheters which penetrate the body, monitoring devices, and underlying conditions, make the patients even more susceptible to these types of infections and increase their risk of occurrence. Pulmonary infections (PI) usually develop after the first 24 hours of ventilator support and they are among the most important complications of these devices; moreover, as high as 20% of mechanically ventilated patients develop PI. 

Several factors such as the presence of underlying diseases (i.e. chronic obstructive pulmonary disease and sepsis), prolonged duration of ventilator support, number of aspirations, etc. play a role in the development of PI (5, 6). Pulmonary conditions, such as pulmonary embolism, pulmonary barotrauma, and pulmonary fibrosis, are other dreaded complications of mechanical ventilation; and the resulting incidence of pulmonary embolism ranges from 7% to 28% (7). Another important complication in these patients is hospital-acquired pneumonia, and this disease is observed in a considerable number of patients under mechanical ventilation. The prevalence of pneumonia in ICU patients is much higher than in other hospitalized patients (5). Prevalence rates of pneumonia increase with prolonged ICU stay; the longer the patient stays in the ICU, the greater the risk of developing hospital-acquired pneumonia (8, 9). In a study conducted during 2005-2006 on 275 patients under mechanical ventilation for 48 hours, 84 subjects (about 30%) developed PI (10). In a cohort study of 361 ICUs, comprising 2897 patients in 20 countries, 439 patients (15%) developed PI. The mortality rate from ventilator-associated pneumonia (VAP) was 38%. The presence of chronic obstructive pulmonary disease, aspiration and sepsis were also recognized as risk factors for this condition (9). In another study done on 98 patients, 53% developed VAP. Factors such as; chronic obstructive pulmonary disease (COPD), and duration of antibiotic therapy, etc. were also influential in the occurrence of VAP (11). Considering the fact that PI is more common in patients under ventilator support and that they are associated with a high mortality rate, studies are required to assess and determine the incidence of this complication, and the factors responsible for its occurrence in order to control and reduce its incidence. To the best of our knowledge, few studies in Iran have evaluated the prevalence of pulmonary infections in patients under mechanical ventilation (MV). 

## 2. Objectives

This study sought to assess the prevalence of PI, related morbidity and mortality, along with predisposing factors in patients with no underlying conditions under MV. 

## 3. Materials and Methods

This descriptive cross-sectional study was conducted on patients hospitalized in the ICU under mechanical ventilation at one hospital; the study was done, from February 2009 to August 2010. ASA class I and II patients with no underlying disease and over 15 years of age, were studied. Patients with an underlying pulmonary disease, immunodeficiency, conditions that increase the risk of infection (i.e. leukemia, chemotherapy, and corticosteroids), and subjects with chronic renal insufficiency requiring ventilator support, were excluded from the study. Thus, patients who required intubation due to a sudden cause (trauma, cerebrovascular accident (CVA), after some surgical procedures, cerebral hemorrhage, etc.), were assessed.

The census method of sampling was used. The ICU attending physician first evaluated the ICU-hospitalized patients, and patients who met the inclusion criteria were included in the study. A total of 40 patients were initially assessed, of whom 37 met the inclusion criteria. The Ethics Committee of the hospital approved the study. Data remained confidential and no change or intervention was made in the patients’ treatment plans. 

Data were recorded in a questionnaire. The questionnaire was designed by the attending physician and it was used to record data on variables such as age, sex, duration of mechanical ventilation, mortality rate, and the number of patients with a pulmonary infection. The first section of the questionnaire was completed with the patients’ demographic information. Patients were examined daily by the ICU physician. In cases developing signs or symptoms of PI, a chest X-ray was obtained and sputum samples (mini-BAL method) were taken and sent for staining and culture. The criterion for a diagnosis of PI was clinical pulmonary infection scores (CPIS) and patients with a score of 6 or higher were considered to have a pulmonary infection and they underwent treatment for VAP (Table 1). Patients with VAP were followed up in terms of response to treatment and recovery, or death due to PI, and the data were recorded in the questionnaire. After data collection in the questionnaires, data were entered in SPSS version 16 software (Statistical Package for Social Sciences, Inc., Chicago, IL). Descriptive statistics were calculated as percentages (%) for qualitative variables and as mean ± standard deviation for quantitative variables. A chi-square test was applied to assess the correlation between qualitative variables. According to normal distribution of data, the t-test was used for comparison of quantitative variables. P < 0.05 was considered statistically significant.

**Table 1.** Clinical Pulmonary Infection Scores ^a^

CPIS Point	0	1	2
**Tracheal tube secretions**	Low	High	High and purulent
**Infiltration on chest x-ray**	No	Diffused	Localized
**Body temperature, ** **˚** **C**	36.5 - 38.5	38.5-38.9	More than 39 or less than 36
**White blood cell count, 1000/mm** ^**3**^	4 - 11	Less than 4 or more than 11	Less than 4 or more than 11 + 500 band cells
**PaO**_**2**_ **/FIO**_**2**_ ** ratio**	Patients with respiratory distress or P/F > 240		Patients with respiratory distress and P/F > 240

^a^ Abbreviations: CPIS, clinical pulmonary infection scores; PaO_2_, partial arterial oxygen tension; FIO_2_, fraction of inspired oxygen.

## 4. Results

This study spanned a period of seven months and it evaluated a total of 37 patients who met the inclusion criteria. The subjects included 21 (56.8%) males and 16 (43.2%) females. The mean age of the patients was 54 ± 19 years (range 19 to 86 years), and the mean age in the males was 52 ± 20 years and 56 ± 18 years in the females (P = 0.52). The mean duration of mechanical ventilation in the patients was 6 ± 4 days (range 2 to 20 days), with 5 ± 2 days for the men, and 6 ± 5 days for the women (P = 0.42). 

**Figure 1.**
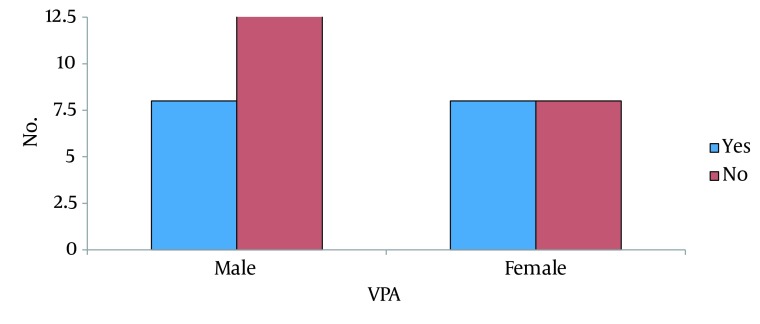
VAP Prevalence Based on Gender

**Figure 2.**
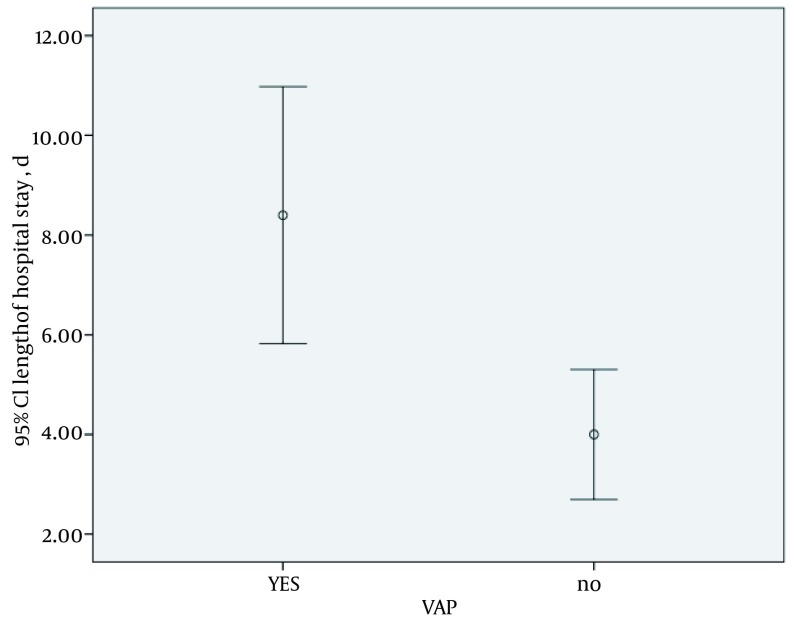
Duration of Mechanical Ventilation in VAP and Non-Affected Patients

**Figure 3.**
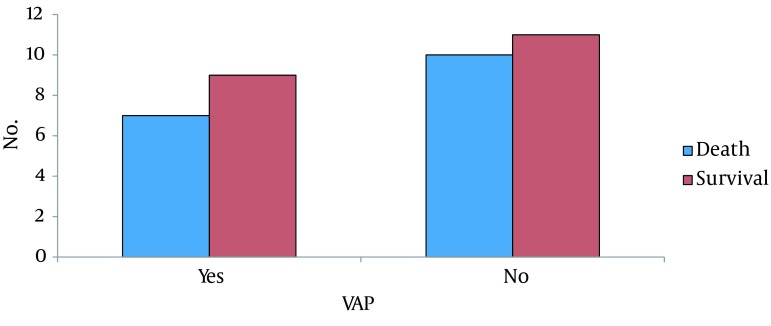
Prevalence of VAP in Survivors and Deceased Patients

Overall, 16 patients (43.2%) developed VAP; of whom, 50% were male and 50% were female (P = 0.46) (Figure 1). Patients who developed VAP had a significantly longer duration of mechanical ventilation. The mean duration of ventilation was 8 ± 4 days in patients with VAP, and 4 ± 2 in non-affected patients (P = 0.005) (Figure 2); 17 patients died; 7 deaths were due to VAP, and 10 were due to other causes (Figure 3). Common microbial pathogens isolated from the sputum of VAP patients were gram-positive cocci, Acinetobacter, gram-negative cocci, Pseudomonas, and Enterobacter, in descending order of frequency. According to an antibiogram, the majority of the previously mentioned microorganisms were susceptible to imipenem and meropenem. Susceptibility to ceftriaxone and cefotaxime was also observed in half of the samples. Ampicillin and penicillin had the highest level of resistance among the microbial pathogens. In five cases, the administered antibiotic was changed due to antibiotic resistance and the most common microorganism responsible in these cases was Pseudomonas.

## 5. Discussion

VAP is common among ICU patients. In this study, approximately half of the patients (43%) developed VAP, of whom 50% were male and 50% were female. This indicates that gender is not a risk factor for VAP. This finding has also been confirmed in previous studies. In our study, the duration of mechanical ventilation had a direct correlation with the prevalence of VAP; which is in accord with previous study results. The majority of patients who developed VAP were under mechanical ventilation for more than six days (mean 8 days). Thus, prolonged duration of ventilation is a serious risk factor for the development of pneumonia. The mortality rate of VAP among all patients in this study was 18.91%; which was lower than the mortality rate in patients deceased due to causes other than pneumonia (27.02%). The majority of these causes was not preventable, including; brain tumors, CVA, and concussions.

Najafi et al. compared the prevalence of VAP between patients with a jejunostomy feeding tube and nasogastric feeding tube. The prevalence of VAP was higher than 65% in patients with a nasogastric feeding tube, while this rate was 13% in those with a jejunostomy feeding tube. The difference between his study and ours is that in our study we excluded patients with underlying conditions or immunodeficiency; therefore, patients had no underlying condition to increase the risk of VAP. However, the prevalence of VAP in our study was still high. A limitation of Najafi’s study is that patients with underlying conditions were not excluded. A limitation of our study we did not assess the patients’ feeding method, and nasogastric feeding per se as a risk factor of VAP (12).

In a study by Noor and Hussain conducted on mechanically ventilated patients in Pakistan, the prevalence of VAP was 28% and the duration of mechanical ventilation correlated with the occurrence of VAP. This finding is in agreement with our results. Another important finding in Noor’s study was that antibiotic administration before intubation correlated with the development of VAP (13).

In a study by Li et al. carried out in trauma patients hospitalized in an ICU in China, the prevalence of VAP was reported to be over 53%. This study was similar to ours in the incidence of PI. On the other hand, the highest prevalence of PI was observed in their study. They also showed that duration of ventilator use was a significant factor in the development of pneumonia (11). In a study by Timsit et al. the mortality rate of ventilator-associated pneumonia was more than 50%. The rate of mortality in their study was greater than that found in our study (14).

In comparison with studies that have been conducted in other countries, the present study showed a relatively high prevalence of VAP in the ICU; however, it should be remembered that our sample size was small because the present study only evaluated patients with no underlying conditions and these patients in the ICU are not many. Moreover, performing certain health and medical procedures, (e.g. intermittent oral rinse with chlorhexidine or normal saline for ventilated patients, placing patients in the head-up position, use of HME antibacterial filters, individualizing certain parts of the ventilator device, for example, connecting pipe or ventilator settings for each patient, and adjusting the pressure of the tracheal tube in the range of 25 -30 mmHg), can reduce the risk of VAP in ventilated patients (15-17). Therefore, these adjustments can reduce the morbidity and mortality in these patients. Unfortunately, none of the above were performed in the present study, and this may have been the cause of the high prevalence of VAP, as well as morbidity and mortality. However, the prevalence rate obtained in our study is lower than that reported by Leone et al. (18). The overall rate of morbidity and mortality in our study was not that high in comparison to other studies, as rates as high as 70% have been reported in some studies. Furthermore, some studies have mentioned old age to be a risk factor, but in our study age had no effect on the prevalence of VAP (which may be due to our small sample size). 

### 5.1. Conclusions

In this study, 43% of ICU patients developed VAP; associated mortality rate of VAP among all of the patients was 18.91%, which was lower than the rate in patients who died from causes other than VAP (27.02%). The duration of mechanical ventilation had a direct correlation with the prevalence of VAP. The most common microbial pathogens isolated from the sputum of patients were gram-positive cocci, Acinetobacter, gram-negative cocci, Pseudomonas and Enterobacter. In an antibiogram, the majority of the above mentioned microorganisms were susceptible to imipenem and meropenem.

### 5.2. Suggestions

The prevalence of VAP in the ICU of this hospital was relatively high compared to other studies and requires further investigation. Thus, it is recommended that sterilization of facilities be carried out with higher efficacy. 
